# Biogas from Macroalgae: is it time to revisit the idea?

**DOI:** 10.1186/1754-6834-5-86

**Published:** 2012-11-27

**Authors:** Adam D Hughes, Maeve S Kelly, Kenneth D Black, Michele S Stanley

**Affiliations:** 1 Scottish Association for Marine Science, Scottish Marine Institute, Department of Ecology, Oban, Argyll, PA37 1QA, Scotland; 2 Scottish Association for Marine Science, Scottish Marine Institute, Marine BioEnergy Scotland, Oban, Argyll, PA37 1QA, Scotland

**Keywords:** Biogas, Methane, Anaerobic digestion, Seaweed, Macroalgae, Aquaculture

## Abstract

The economic and environmental viability of dedicated terrestrial energy crops is in doubt. The production of large scale biomass (macroalgae) for biofuels in the marine environment was first tested in the late 1960’s. The culture attempts failed due to the engineering challenges of farming offshore. However the energy conversion via anaerobic digestion was successful as the biochemical composition of macroalgae makes it an ideal feedstock. The technology for the mass production of macroalgae has developed principally in China and Asia over the last 50 years to such a degree that it is now the single largest product of aquaculture. There has also been significant technology transfer and macroalgal cultivation is now well tried and tested in Europe and America. The inherent advantage of production of biofuel feedstock in the marine environment is that it does not compete with food production for land or fresh water. Here we revisit the idea of the large scale cultivation of macroalgae at sea for subsequent anaerobic digestion to produce biogas as a source of renewable energy, using a European case study as an example.

## Introduction

Growing terrestrial crops for biofuel may make a negligible contribution to net greenhouse gas emissions [1,2] and may cause other environmental impacts while reducing freshwater resources and food security [3]. Given these limitations there has been renewed / increased interest in aquatic and marine production for biofuels [4,5]. This interest can be divided into two principal components: biofuels derived from macroalgae (seaweed) and biofuels derived from microalgae (single cell plants). Microalgal derived biofuels have received much attention as a source for biodiesel [6-8], however production costs are an order of magnitude too expensive [3]. Although there is currently enormous research investment into the bulk production of microalgae for biodiesel, photo bioreactors are unlikely to be economically competitive for bioenergy production, and culture in outdoor ponds is only suited to regions with a relatively high number of sunlight hours and even then may still be uncompetitive in the biofuels market [9].

Macroalgae as a source of bioenergy first received intensive scrutiny as part of the US Ocean Food and Energy Farm project as proposed by Wilcox [10], initiated in 1973 and lasting over a decade [11]. It resulted in the construction of ocean farms for cultivation of the giant kelp *Macrocystis*[12]; reviewed by Kelly and Dworjanyn, [13]. While farming this species of seaweed in this truly offshore environment presented many technical challenges, the biogasification of macroalgal biomass gave excellent results [10,12,14,15]. This and subsequent research highlights some of the major advantages of macroalgae over other sources of biofuels (see Table 1).

**Table 1:** **Environmental and societal risk associated with terrestrial biofuels (after Koh and Ghazoul, **[16]**) and macroalgae biofuels**

**Environmental and societal advantages of macroalgae production for biofuels**	
Net GHG emissions from land-use change	The culture of macroalgae for biofuel would be entirely marine based and would not have the associated GHG emissions associated with land use change.
Threats to biodiversity	Macroalgae cultivation takes place in the water column above the seabed. Impacts of large scale macroalgae production on benthic biodiversity are currently unquantified. Likely impacts will include shading and competition for nutrients. However, most production will be in waters where the seabed is deeper than the photic zone, and where terrestrial nutrient run off creates hypernutrified water. It is likely that biodiversity would increase in the vicinity of macroalgae farms as a result of increased habitat structural complexity.
Impacts on food prices	Currently most macroalgae cultivation is for human consumption. Large scale production of macroalgae for biofuels is bound to distort this market. However the impacts on the supply of macroalgae to human food chain is likely to be small due to a clear market segregation and the far higher value of macroalgae as food compared to the price of energy.
Competition for water resources	Mass cultivation of macroalgae has a zero freshwater requirement and only modest amounts are required in anaerobic digestion

With microalgae much of the research interest has focused on their conversion to liquid biofuels such as ethanol [17-20]. However, in this review we focus on anaerobic digestion of cultivated macroalgae for the production of biogas. Since this original gasification / culture research was conducted there have been substantial advances in macroalgal cultivation and offshore engineering. However the concept of ocean farming for biogas production has received relatively little attention in the 21^st^ century.

**Figure 1:**
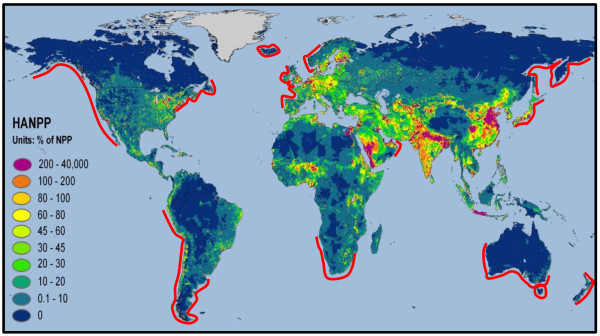
**Natural distribution of shallow water macroalgae (red line) indicating coastal areas with the potential to culture macroalgae for biogas, and human appropriation of net primary production as a percentage of local net primary productivity (NPP) (NASA 2004 **[30]**). ** Redrawn from Santelices (2007) [31] and NASA Earth Observatory 2004.

In coastal temperate regions this technology may have significant potential to meet local energy demand, particularly in areas where the expansion of terrestrial biofuel production is limited due to high percentages of net primary productivity (NPP) already being appropriated for human use, such as the north Atlantic Coast of North America and Europe, and the western seaboard of South America (Figure 1). In this review the wild harvest of seaweeds for biofuel is not considered a viable option either in terms of potential yield or its environmental impact. Although macroalgal harvest for high value products takes place in some countries, very careful management is required to prevent serious ecosystem damage [21-24] hence it would be impossible to justify harvest on the massive scale necessary [25-27] to make a significant energy contribution. Macroalgal forests, as with other biogenic structures in the marine environment such as corals and seagrasses, are considered to be biodiversity hot spots providing important habitat to a wide range of organisms including fish and birds [28]. It is worth noting that in Norway where approximately 170,000 tonnes pa *Laminaria hyperborea* are harvested, even a 4–5 year rotation is not always sufficient to allow recovery [29]. In addition, as wild stocks are generally dispersed around coastlines this would result in high costs of transport to processing plants.

### Macroalgal culture

Currently over 100 species of macroalgae are used for food, in medicine, or as fertiliser and in the processing of phycolloids and chemicals [32]. Although used for millennia, their domestication only began in the twentieth century as a fuller understanding of their life cycle was achieved [33]. Several species are now in culture on a large-scale in east Asia. China is the world’s largest producer of cultivated seaweed, mostly grown on long-line systems where hatchery produced seedlings are transplanted to sea on ropes suspended vertically from a horizontal top-line. The large brown *L. japonica* known as haidai or ‘sea-strap’ and originally introduced to China from Japan is the world’s most cultivated species by volume and value. It was the first seaweed to be subjected to the entire process of seeding, tending and planting out and to have the status of a marine plant crop [32]. Global production of *L. japonica* alone in 2010 was 5.14 million tonnes with a value of 3.01 billion USD [34]; it is grown primarily for food but also for iodine and alginates. Its fast growth and high productivity make this and several other species of brown macroalgae particularly suited to culture for energy crops. Estimates of macroalgal primary productivity rates, in terms of carbon capture during photosynthesis, are approximately 1600 g Cm^-2^y^-1^[35], comparing favourably to a global net primary productivity of crop land of 470 g Cm^-2^y^-1^[36].

Selective breeding of macroalgae began in China in the 1960s with *Laminaria* species. This has resulted in a number of varieties that show the enhancement of desirable characteristics over wild varieties. These characteristics include:

i. increased frond growth rate at higher temperatures, resulting in a longer frond and higher production (20-58% higher)

ii. a higher (8-40%) iodine content as compared with the natural population

iii. a lower water content

Since the early seventies these selectively bred strains have been widely adopted by the *Laminaria* cultivation industry in North China. There are thus good prospects for the development of strains having traits desirable for biofuel production, such as increased sugar content or altered seasonality of production cycles [37].

### Macroalgal production

In Europe, hatchery raised macroalgae have been cultured successfully on long-line systems, similar to those used for mussel production. Positioned adjacent to salmon cages in Scottish sea lochs [38], a 100 m horizontal long-line bearing vertical strings carrying seaweeds every 50 cm, indicated average yields of >50 kg (native *Saccharina latissima*) per horizontal meter of long-line. If this were extrapolated to consider 40 such 100 m longlines, then yields of 200 t wet weight ha^-1^ (approximately 20 t dry weight) would be obtainable. This is comparable to yields achieved in China without fertiliser (H. Liu pers.comm. citing China Fish Annals, 2003). However if macroalgal crops are to make a significant contribution to fuel supply then very large areas would have to be farmed. MacKay (2009) [39] makes it clear that biomass energy will need to be a country-scale activity to make a meaningful contribution to UK energy needs. This will require significant changes in societal attitudes to use of the marine environment and, in many countries, regulatory changes. Inshore areas are already under significant pressure so the culture of macroalgae at the scale required for biofuel production must be largely located on continental shelves. Globally there is a very large amount of continental shelf suitable for such a massive aquaculture expansion; presently aquaculture occupies only about 0.04% of continental shelf area [40]. However culturing seaweed in an European offshore environment will require the development of more mechanised technologies for outplanting and harvest than the labour intensive methods on which the large-scale culture in Asia currently depends. This in turn may lead to the development of more specialised vessels than the mussel/salmon-farm work boats currently employed. The growth rate and productivity of seaweeds, grown on a large and dense scale, and in a different nutrient regime (offshore) to that of the inshore waters (Scottish sea lochs) has yet to be verified.

### Seaweed to biogas: anaerobic digestion

Macroalgae can be converted to biofuels by various processes including thermal treatment [41] and fermentation [19,42] but the most direct route to obtaining biofuel from macroalgae is via its anaerobic digestion (AD) to biogas (~ 60% methane). Methane can be used to produce heat and electricity or compressed for use as a transport fuel. Research conducted in the 1980’s [43,44] still provides a bench mark for biogas yields for a number of macroalgal species, but since this time there have been developments in AD technology and an enormous increase in its use.

In comparison to terrestrial biomass crops, macroalgae contain little cellulose and no lignin and therefore undergo a more complete hydrolysis. Gas yield is related both to ash content (and its inverse relationship with volatile solids content) and the level of storage sugars; and, as seaweed biochemical composition varies with season, gas yield will vary [45,46]. The C:N ratio is also an important part of optimising digester diet and strengthens the argument for the co-digestion of seaweeds with other more N rich substrates, for example waste food or agricultural slurries. Biogas yields are also dependent on a wide range of other variables such as inoculum, digester system configuration and feed stock composition.

**Figure 2:**
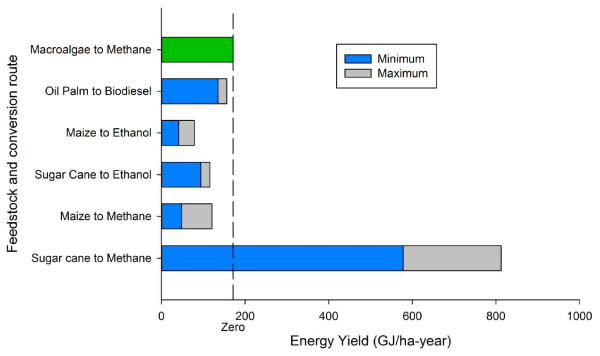
**The energy production of biofuel crops ha**^**-1 **^**based on macroalgal production of 200 t ww ha**^**-1**^**, terrestrial biofuel crop estimates are from Shilton and Guieysse (2010) **[3]**.**

Perhaps the most realistic estimate of the true industrial potential of methane production from macroalgae were obtained by Matusi (2006) [47] using a commercial scale 4 stage anaerobic digester for over 150 days, with a daily input between 0.2-1.0 tonnes of seaweed and a retention time of 15 to 25 days. This resulted in an average production of 22 m^3^ of methane per tonne wet weight of brown seaweed (*Laminaria* sp). The potential energy yield from the AD of marine biomass compares favourably with that of terrestrial crops (Figure 2) on an energy per unit area basis. These figures are based on a production of 22 m^3^ of methane per tonne of macroalgae wet weight, and a production of 200 tonnes of macroalgae ha ^-1^. However recent advances suggest there is still potential for further optimising biogas yields through co-digestion with a more nitrogenous substrate [48,49] and manipulation of the microbial composition of the inoculum [50].

### Bioenergy potential – a question of scale

If we use a realistic estimate of macroalgal production [26] (200 t ha ^-1^) and a conservative estimates of biogas yield after conversion (22 m^3^ tonne wet weight (ww)) yielding 171 GJ ha ^-1^ we can see that to make a significant contribution to bioenergy targets there will need to be macroalgal cultivation on a massive and unprecedented scale. For example if all of the brown algae currently produced in culture (6.8 million tonnes p.a. [34]) was converted to biogas using the parameters above it would yield approximately 5.7 PJ which is approx. 0.06% of the UK total energy demand for 2010 (9518 PJ [51]). To meet 1% of UK total energy demand would require an area of cultivation of approximately 5440 km^2^. This is equivalent to half of the entire global area currently used for aquaculture production. However, if this is put in context of available space, this area accounts for only approximately 3% of the UK territorial waters (161200 km^2^). By comparison with terrestrial biofuel production in the UK, to produce 1% of the UK’s total energy demand using maize to methane would require a land area of 7700 km^2^, equivalent to 18% of the UK’s cropland (45000 km^2^[52]). Although neither scenario seems attractive, such comparisons clearly illustrate the potential advantages of scale in moving UK biofuel production into the marine environment.

**Figure 3:**
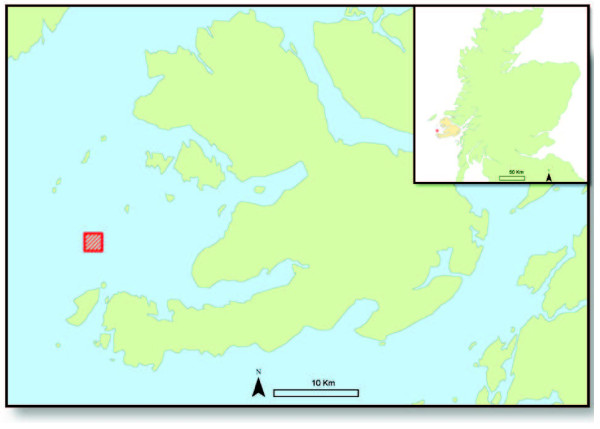
Area needed to grow enough seaweed to meet domestic gas requirements for homes on the Isle of Mull, West coast of Scotland, based on a production of 200 wet tonnes ha −1.

At a regional level large-scale macroalgal culture for biofuels offers real potential for rural coastal communities. A good example would be for the Isle of Mull on the west coast of Scotland which has no domestic gas supply, the main fuels being heating oil and electricity. The Isle of Mull has 1278 households [53] and the average UK annual domestic gas consumption is 57.6 GJ [54], so to provide all the households on Mull with gas would require the methane from 430 ha of macroalgae cultivation, the extent of which can be seen on Figure 3. We envisage this would both increase local employment and improve rural fuel security. The cultivation of macroalgae for biofuels could be developed through a modular approach in a European/American context where the distances between culture, processing and AD facilities are minimised, similar to that proposed for Japan [55]. Seaweed farms would supply local, coastal processing facilities where high value products can be first extracted from the crop before transfer to an on-site or a shared AD facility. From here the biogas produced could be piped directly to augment the local natural gas supply.

### Environmental impact

Environmental impacts of large scale seaweed farms may arise from; changes to local hydrodynamics and resulting sedimentation patterns, benthic impacts from increased organic matter supply, changes to water column nutrient availability and from shading of the sea-floor (in shallow sites). Although we anticipate some types of interactions may well be positive [25] a measure of the extent and nature of interactions with fish, cetaceans and birds as well as other users of the marine environment for aquaculture, fisheries, energy generation and shipping is required.

During the growth cycle a portion of the macroalgae and the associated biota from the culture lines will be lost to the benthos either through erosion of the blade tips or shearing of cultured material creating an organically enriched zone [56]. In ‘fed’ aquaculture, that of fin-fish for example, where high energy feeds are supplied to the system, measurement of the extent of the zone of deposition is required [49] and has in turn led to the development of regulatory tools [57]. Although the macroalgal cultures are not ‘fed’ i.e. artificially supplied with additional nutrients or fertilised, the extent and effect of the zone of organic enrichment should be described. In enclosed water bodies, there may be competition for dissolved nutrients with phytoplankton but, in more open shelf systems, nutrient supply is likely to be sufficient provided that farms are spatially arranged for optimal nutrient exchange. In any event, nutrients taken up by macroalgal culture, on the scale required for biofuel production, would be far less than that produced by agricultural, urban sources and fin-fish aquaculture. If macroalgae is subjected to the AD process then a proportion of the nitrogen may be lost through denitrification depending on the conditions in the reactor. Digestates are typically higher in ammonia and lower in organic nitrogen than ingestates [58]. The digestate will most likely be used in fertilisers and so find its way back into the hydrological cycle.

There may also be a number of positive benefits; the macroalgal farms effectively acting as no-take zones for mobile gear fisheries and thus enhancing less destructive static gear fisheries within the cultivation zone and providing spill over benefits to adjacent waters [59]. In addition, providing the crop is not removed in its entirety at the end of the cycle it will provide a refuge and a substrate to enhance local biodiversity. The digestate after AD may be either a valuable by-product or an expensive waste. This will depend on a number of factors including its contaminant metal burden and whether the macroalgae has been mixed with other organic waste streams in the digestor. A study on the AD of lipid-extracted microalgal biomass [60] suggested that 80% of the nitrogen in the biomass was recoverable as ammonium/ammonia from the liquid supernatant fraction, and that the remaining nitrogen in the solid digestate fraction had a 40% bioavailability when applied to soil. A similarly detailed analysis of the fate of nitrogenous emissions following AD of macroalgal biomass is required. Overall the global effect of using macroalgal culture for biofuel is likely to be positive and an initial full life cycle analysis of biomethane production from offshore cultivation of macroalgae has shown a 69% reduction in fossil fuel utilisation when compared to natural gas, a 54% reduction in greenhouse gas emissions and an improvement in the marine eutrophication index [61].

### Making it pay

Costing the culture of large amounts of seaweed in a European context is currently highly uncertain as there are too many unconstrained parameters, such as scalability, location and the degree of mechanisation readily achievable. However, our analysis based on inshore production suggest that at 2011 wellhead value for natural gas (US $3.95 [62] per thousand cubic feet (equivalent to £0.09 m^3^)) based on a production of 20 tonnes dry weight (dw) ha ^-1^ the production costs for macroalgal biogas would have to be less than £400 ha ^-1^ to be competitive with fossil fuels without additional subsidy. It is unlikely that in the short term such production costs could be achieved. However under the UK Renewable Heat Incentive 2011 [63] scheme injection of biomethane into the natural gas grid attracts a price of £0.068 kWh. This is equivalent to £3230 hectare which would make the cultivation of macroalgae for methane production highly competitive. In addition the identification and extraction of higher value products, prior to AD, is advisable, as is the quantification of how the prior extraction affects biogas yield. Added value could be achieved by processing part of the crop for human and animal foodstuffs, and food supplements, for its mineral content for animal feeds, as an organic slow release fertiliser, and potential bio-active compounds [64].

## Conclusion

Our analysis of growth data from hatchery-raised macroalgal sporelings outplanted to conventional long-line systems in Scotland suggests there are no major biological obstacles to the culture process in a European context. A fuller understanding of the impacts and performance of native macroalgae grown in dense large-scale cultures can only be achieved through pilot scale trials. Technological advancement is required to mechanise the outplanting and harvest process. The biological gasification of macroalgae was well proven in the later decades of the 20^th^ century and AD technology has sufficiently matured to offer a range of possibilities to further optimise methane yields. Compared to first generation biofuels, macroalgae have inherent advantages that make them environmentally sustainable. Given that fossil fuel prices are likely to increase and that macroalgal production costs will inevitably fall as production is expanded and intensified, it is prudent to develop the technology required to obtain significant quantities of biofuel from marine biomass in time to help meet Europe’s energy needs and climate change targets.

## Abbreviations

AD: Anaerobic digestion; C: Carbon; dw: Dry weight; GHG: Greenhouse gas; GJ: Giga joules; ha: Hectare; hr: Hour; kg: Kilogram; km: Kilometre; kW: Kilowatt; m: Meter; N: Nitrogen; NPP: Net primary productivity; pa: Per annum; PJ: Peta joules; Sp: Species; t: Tonne; UK: United Kingdom; US: United States; ww: Wet weight.

## Competing interests

The authors declare that they have no competing interests.

## Authors’ contributions

This review was conceived, researched and written by ADH, MSK, KDB and MSS. All authors have read and approved the final manuscript.
